# P-1695. Antimicrobial prescribing differences for urinary tract infections between advanced practice practitioners and physicians at a safety-net community hospital

**DOI:** 10.1093/ofid/ofae631.1861

**Published:** 2025-01-29

**Authors:** Jessica Hua, Aakash Balaji, Ben Pomerantz, Dylan Huber, Lawrence Sanchez, Mirza Ali, Alfredo J Mena Lora

**Affiliations:** University of Illinois at Chicago, Chicago, Illinois; University of Illinois, Chicago, Illinois; University of Illinois at Chicago, Chicago, Illinois; Saint Anthony Hospital, Chicago, Illinois; Saint Anthony Hospital, Chicago, Illinois; Saint Anthony Hospital, Chicago, Illinois; University of Illinois Chicago, Chicago, Illinois

## Abstract

**Background:**

Advanced practice practitioners (APPs) are increasingly working in inptient settings as hospitalists. Despite these trends, there's a paucity of data on APP antimicrobial prescribing practices. Antimicrobial stewardship programs (ASPs) can reduce antimicrobial overuse and misuse. One of the most common infections in the inpatient setting is urinary tract infections (UTIs). We examine antibiotic prescribing practices of APPs and physicians for treatment ofUTIs. Understanding prescribing practices can highlihgt stewardship gaps and help guide ASP efforts.

Figure 1

**Figure:**
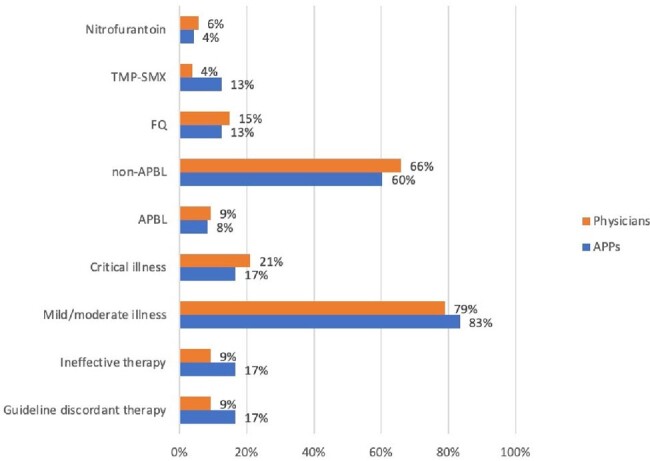

UTI severity and empiric antimicrobial selection by physicians and APPs

**Methods:**

A retrospective analysis of antimicrobial prescriptions was conducted at a 151-bed community hospital. ASP prospective audit and feedback (PAF) data for UTIs from July 2022 to June 2023 was reviewed, including initial antimicrobial selection, duration, and guideline concordance for APPs and physicians. Guideline adherence, antimicrobial effectiveness based on local antibiogram, and days of therapy (DOT) per 1000 patient days was compared between APPs and physicians.

Figure 2

**Figure:**
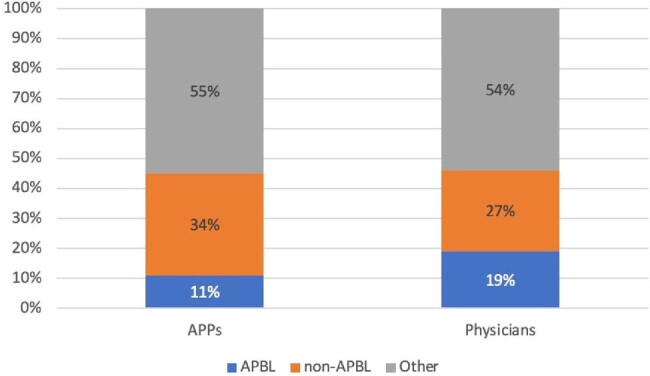

Empiric antimicrobial selection by physicians and APPs

**Results:**

A total of 209 initial PAF events for UTIs were reviewed, of which, 161 were physicians and 48were APPs. Physicians’ initial empiric choice was 9% (15) guideline discordant and ineffective. APP initial choice was 17% (8) guideline discordant and ineffective. TMP-SMX was more commonly used by APPs (Figure 1). Total DOT/1000 was 145. DOT/1000 was 44 for physicians, of which 11% were anti-pseudomonal beta lactams (APBLs) and 34% non-APBLs. DOT/1000 was 21 for APPs, of which 19% were APBLs and 27% non-APBLs (Figure 2). DOT/1000 was 44 for physicians, of which 11% were anti-pseudomonal beta lactams (APBLs) and 34% non-APBLs. DOT/1000 was 21 for APPs, of which 19% were anti-pseudomonal beta lactams (APBLs) and 27% non-APBLs (Figure 2).

**Conclusion:**

APPs more commonly chose guideline discordant or ineffective initial empiric choices for UTIs compared to physicians. Empiric selection of ineffective agents according to local antibiogramswas higher for APPs. This underscores the importance of targeted ASP education on UTI guidelines and local antibiogram interpretation to improve antibiotic prescribing practices among APPs.

**Disclosures:**

**All Authors**: No reported disclosures

